# USP9X-mediated REV1 deubiquitination promotes lung cancer radioresistance via the action of REV1 as a Rad18 molecular scaffold for cystathionine γ-lyase

**DOI:** 10.1186/s12929-024-01044-3

**Published:** 2024-05-28

**Authors:** Yunshang Chen, Xue Feng, Zilong Wu, Yongqiang Yang, Xinrui Rao, Rui Meng, Sheng Zhang, Xiaorong Dong, Shuangbing Xu, Gang Wu, Xiaohua Jie

**Affiliations:** 1 Cancer Center, Union Hospital, Tongji Medical College, Huazhong University of Science and Technology, Wuhan, 430022 China; 2 Institute of Radiation Oncology, Union Hospital, Tongji Medical College, Huazhong University of Science and Technology, Wuhan, 430022 China; 3 Hubei Key Laboratory of Precision Radiation Oncology, Wuhan, 430022 China; 4 Department of Obstetrics and Gynecology, Union Hospital, Tongji Medical College, Huazhong University of Science and Technology, Wuhan, 430022 China

**Keywords:** Non-small cell lung cancer, Radioresistance, REV1, USP9X, Amino acid metabolism

## Abstract

**Background:**

Radioresistance is a key clinical constraint on the efficacy of radiotherapy in lung cancer patients. REV1 DNA directed polymerase (REV1) plays an important role in repairing DNA damage and maintaining genomic stability. However, its role in the resistance to radiotherapy in lung cancer is not clear. This study aims to clarify the role of REV1 in lung cancer radioresistance, identify the intrinsic mechanisms involved, and provide a theoretical basis for the clinical translation of this new target for lung cancer treatment.

**Methods:**

The effect of targeting REV1 on the radiosensitivity was verified by in vivo and in vitro experiments. RNA sequencing (RNA-seq) combined with nontargeted metabolomics analysis was used to explore the downstream targets of REV1. Liquid chromatography-tandem mass spectrometry (LC-MS/MS) was used to quantify the content of specific amino acids. The coimmunoprecipitation (co-IP) and GST pull-down assays were used to validate the interaction between proteins. A ubiquitination library screening system was constructed to investigate the regulatory proteins upstream of REV1.

**Results:**

Targeting REV1 could enhance the radiosensitivity in vivo, while this effect was not obvious in vitro. RNA sequencing combined with nontargeted metabolomics revealed that the difference result was related to metabolism, and that the expression of glycine, serine, and threonine (Gly/Ser/Thr) metabolism signaling pathways was downregulated following REV1 knockdown. LC-MS/MS demonstrated that REV1 knockdown results in reduced levels of these three amino acids and that cystathionine γ-lyase (CTH) was the key to its function. REV1 enhances the interaction of CTH with the E3 ubiquitin ligase Rad18 and promotes ubiquitination degradation of CTH by Rad18. Screening of the ubiquitination compound library revealed that the ubiquitin-specific peptidase 9 X-linked (USP9X) is the upstream regulatory protein of REV1 by the ubiquitin-proteasome system, which remodels the intracellular Gly/Ser/Thr metabolism.

**Conclusion:**

USP9X mediates the deubiquitination of REV1, and aberrantly expressed REV1 acts as a scaffolding protein to assist Rad18 in interacting with CTH, promoting the ubiquitination and degradation of CTH and inducing remodeling of the Gly/Ser/Thr metabolism, which leads to radioresistance. A novel inhibitor of REV1, JH-RE-06, was shown to enhance lung cancer cell radiosensitivity, with good prospects for clinical translation.

**Supplementary Information:**

The online version contains supplementary material available at 10.1186/s12929-024-01044-3.

## Introduction

The genomes of all organisms are under constant attack by endogenous and exogenous genotoxic factors causing DNA damage, such as ultraviolet light, ionizing radiation, and substances generated through metabolic processes, and organisms have evolved multiple DNA damage repair pathways to protect genome integrity [1–3]. Translesion DNA synthesis (TLS) is a form of DNA damage tolerance conserved in prokaryotes and eukaryotes that utilizes a specific set of low-fidelity TLS polymerases to complete DNA replication in a manner that preserves DNA damage rather than repairs [4, 5]. Our previous study found that the expression of REV1 DNA directed polymerase (REV1), a core member of the TLS polymerase family, was significantly upregulated in lung cancer tissues and correlated with poor prognosis. REV1 promotes lung tumorigenesis by activating the Rad18/SERTAD2 axis [6]. This indicates that REV1 can biologically function as an oncogene in lung cancer. Of interest, recent studies have confirmed the important role of REV1 in DNA damage repair and the maintenance of genomic stability. In saccharomyces cerevisiae, cells lacking REV1 are sensitive to DNA damage agents, including cisplatin, mitomycin C and UV radiation [7]. Similarly, REV1 was found to play an important role in the activation of the ATR-Chk1 checkpoint during mitomycin C-induced interstrand crosslink repair in African clawed toad egg extracts [8]. In mammalian cells, REV1 deficiency leads to increased oxidative stress, cellular senescence, and apoptosis. Elevated REV1 expression enhances cellular tolerance to UV damage. High levels of REV1 can increase the frequency of gene mutations in cells, thus helping them to adapt to environmental stress [7, 9, 10]. Radiotherapy is a cytotoxic therapy that exerts its therapeutic effect mainly by inducing double-stranded DNA breaks in tumor cells, and DNA damage repair is an important factor affecting tumor radiosensitivity [2, 11]. Therefore, REV1 may be closely related to radioresistance in tumor cells; however, the role of REV1 and regulatory mechanisms through which it functions in tumor radioresistance have not been elucidated.

Cancer cells undergo metabolic reprogramming to support a higher demand for energy and metabolites for continued cell growth and proliferation [12, 13]. Radiation can affect cellular metabolism, and adaptive metabolic reprogramming of cancer cells can also affect the efficacy of radiotherapy [14]. For example, postirradiation tumor cells can undergo metabolic reprogramming by upregulating glutamine synthetase (GS) expression, and abnormally expressed GS promotes glutamine synthesis from glutamate, causing enhanced nucleotide metabolism and accelerating the DNA damage repair process, thereby inducing resistance to radiotherapy [14, 15]. Activation of serine/glycine synthesis is associated with radioresistance by protecting cells from oxidative stress, and inhibition of serine/glycine synthesis may impair the formation of new blood vessels in the tumor microenvironment, leading to tumor hypoxia and thus enhancing the response to radiotherapy [16]. In addition, targeting ab initio serine/glycine biosynthesis improves the antitumor efficacy of radiotherapy by inhibiting the M2 polarization of macrophages [17]. Cystathionine γ-lyase (CTH) is a cytoplasmic enzyme involved in the catabolism of glycine, serine and threonine (Gly/Ser/Thr) by mediating the conversion of methionine-derived cystathionine to cysteine. Xia et al. reported that CTH affects intracellular glutathione (GSH) synthesis and reactive oxygen species (ROS) content by regulating amino acid metabolism, indicating that CTH may be related to tumor radiosensitivity [18]. The above studies suggest that targeting cellular metabolic remodeling, especially targeting amino acid metabolism, is expected to improve tumor response to radiotherapy.

In this study, we report for the first time that the high REV1 expression in lung cancer is associated with the deubiquitinase USP9X and that abnormally high REV1 expression can act as a scaffolding protein to assist the E3 ubiquitin ligase Rad18 binding to the substrate CTH. This binding promotes the ubiquitin-mediated degradation of CTH, thereby increasing the intra- and extracellular levels of Gly/Ser/Thr and inducing the formation of an abnormal tumor metabolic microenvironment that ultimately renders lung cancer cells insensitive to radiotherapy. Genetic or pharmacological inhibition of REV1 can induce metabolic reprogramming in tumor cells and ultimately reverse radiotherapy resistance in lung cancer. Our results suggest that REV1 is a promising therapeutic target, and its inhibitor JH-RE-06 is expected to be used as a novel radiosensitizer for clinical applications.

## Materials and methods

### Cell lines

Human lung cancer cells A549, H1299 and H1975 were purchased from the American Type Culture Collection and cultured according to the manufacturer’s instructions. All cells were tested for cell identification and mycoplasma contamination before use. Lung cancer cell lines with stable REV1 knockdown were constructed according to a previously described method [6]. Transfection of small interfering RNAs (siRNAs) was performed using a transfection reagent (Lipofectamine RNAiMAX, Invitrogen) following the steps in the instructions. The shRNA and siRNA sequences used in this study are given in Supplementary Table S1.

### Antibodies and reagents

The main antibodies and reagents used in this study are as follows: mouse anti-REV1 (Santa Cruz, sc-393022, 1:100), rabbit anti-USP9X (Proteintech, 55054-1-AP, 1:1000), rabbit anti-CTH (Abcam. ab151769, 1:1000), mouse anti-GAPDH (ABclonal, AC033, 1:3000), rabbit anti-Rad51 (Abcam, ab133534, 1:1000), rabbit anti-USP24 (Proteintech, 13126 -1-AP, 1:1000), rabbit anti-USP5 (Proteintech, 10473-1-AP, 1:1000), rabbit anti-USP1 (Proteintech, 14346-1-AP, 1:1000), rabbit anti-USP14 ( Proteintech, 14517-1-AP, 1:1000), rabbit anti-UCHL5 (Proteintech, 11527-1-AP, 1:1000), mouse anti-Flag (Sigma-Aldrich, F1804, 1:3000), rabbit anti-GST (Abcolonal, AE006, 1:1000), rabbit anti-HA (Cell Signaling Technology, #3724, 1:3000), Ubiquitination Compound Library (Selleck, L6000), JH-RE-06 (Topscience, T15611), MG132 (Calbiochem, 474,790) and cycloheximide (Sigma-Aldrich, A8244).

### Western blotting and immunoprecipitation

Western blotting was performed according to the standard procedure in our previously published article [19–21]. For exogenous immunoprecipitation analysis, the treated cell supernatants were incubated with S beads overnight at a low temperature (4 °C) and the next day were repeatedly washed with NETN buffer 5 times, followed by Western blotting. For endogenous immunoprecipitation experiments, treated cell lysates were incubated with the corresponding antibodies for 1 h at low temperature, and then A/G beads were added and incubated overnight; the next day the beads underwent 5 repeated washes with NETN buffer the next day followed by Western blotting analysis.

### GST pull-down assay

The GST-vector and GST-CTH plasmids were transformed into *E.coli* BL21, and the bacteria were cultured in LB-ampicillin overnight on a shaking incubator at 37 ℃. When the optical density of the bacterial solution reached 0.8, β-d-1-thiogalactopyranoside was added to induce protein expression. Bacteria were collected by centrifugation, and GST-only and GST-CTH fusion proteins were harvested by using Bacterial Protein Extraction Kit (Boxbio, AKPR020-2) according to the manufacturer’s protocol. Flag-Rad18 plasmid was transfected in HEK293T cells, and the cell lysates were collected 24 h later and incubated with GST-only or GST-CTH fusion proteins plus GST beads (GE Healthcare) at 4 ℃ overnight. The beads were repeatedly washed with NETN buffer for 5 times on the next day followed by Western blotting analysis.

### In vivo ubiquitination assay and protein half-life assay

For the ubiquitination assay, the experimental steps were as follows: USP9X knockdown lung cancer cells were cotransfected with 2 µg each of the SFB-REV1 and HA-ubiquitin plasmids, and the medium was changed after 4-6 h. The plasmids were transfected for 24 h and then the cells were treated with the proteasome inhibitor MG132 for 4 h. The cells were collected, and the proteins were extracted, and 0.5-1 mg samples of each were subjected to co-IP with S beads, followed by Western blotting to detect HA, and the ubiquitination level of REV1 protein was analyzed. For protein half-life experiments, after receiving the corresponding treatment, cells were treated with cycloheximide (final concentration of 20 µg/ml) for various times. Then, cells were collected, proteins were extracted, and the expression levels of proteins at different time points were detected by Western blotting to analyze the effect of USP9X on REV1 half-life.

### RNA sequencing and analysis

Total RNA was isolated and extracted using an AFTSpin Cell Fast RNA Extraction Kit (RK30120), and all samples were sequenced by BGI-Shenzhen (China). The reference genome GCF_000001635.26_GRCm38.p6 was used as a control for HISAT comparison to identify differentially expressed genes for subsequent analysis. The RNA sequencing data generated in this study have been uploaded to the GEO database (GSE183332).

### Metabolomic sequencing

In this study, untargeted metabolomics sequencing using LC-MS/MS technology was performed by BGI-Shenzhen (China). A high-resolution mass spectrometer Q Exactive HF (Thermo Fisher Scientific, USA) was used to acquire data in both positive and negative ion modes to improve metabolite coverage. Data processing was performed using Compound Discoverer 3.1 (Thermo Fisher Scientific, USA) software. The raw multivariate data were downscaled by principal component analysis (PCA), and partial least squares method-discriminant analysis (PLS-DA) was used to obtain the fold change from univariate analysis (fold change). Student’s t test results obtained from univariate analysis were used to screen differential metabolites.

### Real-time quantitative polymerase chain reaction (qRT-PCR)

Total RNA was isolated and extracted using an AFTSpin Cell Fast RNA Extraction Kit (RK30120). cDNA was obtained by reverse transcription using ABScript III RT Premix (RK20428) and assayed according to Genious 2x SYBR Green Fast qPCR Mix (RK21204). The manufacturer’s online instructions were followed to perform the assay, and the relative target gene mRNA levels were calculated using the ΔΔCt method. Primer sequences are given in Supplementary Table S2.

### Cell clonogenic survival assay

The cells were irradiated with different doses of X-rays as described before and incubated for 7-14 days. The medium was discarded and cells were washed twice with PBS, fixed with 4% paraformaldehyde for 15 min, stained with crystal violet for 30 min, washed and dried naturally. The number of clones was counted under a microscope (≥ 50 cells were considered positive clones), and the colony formation curve was plotted [19, 22].

### Neutral comet assay

This assay was used to detect alterations in trailing comet formation in lung cancer cells after radiotherapy to assess the extent of DNA damage. The Trevigen kit (TRE-4250-050-K) was used for this assay, following the standard procedure described in our previously published article [19]. In brief, treated cells were mixed proportionally with Comet LMAgarose and evenly coated in Comet Slide™ sample wells, the slides were then cooled, and immersed in Lysis Solution for 1 h, after which the slides were immersed in prechilled 1× Neutral Electrophoresis Buffer for 30 min, followed by the application of constant pressure after electrophoresis. The slides were immersed in DNA Precipitation Solution for 30 min at room temperature, and each well was incubated with 100-150 µL of SYBR Gold (Invitrogen) staining solution for 8 min at room temperature for staining. Then, the slides were washed with triple distilled water to remove excess staining solution and dried overnight in a light-proof box. All operations were performed at 4 °C and with light avoidance unless otherwise indicated. Fluorescence microscopy was used for observation and imaging, and the results were analyzed using CometScore 2.0 and GraphPad Prism 8 software.

### Immunofluorescence staining

This experiment was used to detect alterations in the DNA repair capacity of lung cancer cells after radiotherapy. It was performed according to the standard procedure described in our previously published article [19, 23]. In brief, treated cells were fixed with 4% paraformaldehyde, lysed with 0.2% Triton X-100, and incubated with an anti-Rad51 antibody overnight at 4 °C. The next day, a fluorescent secondary antibody was added, the slides were removed, and the slides were inverted to treat the cells with an anti-fluorescence quencher. The changes in Rad51 foci were detected by laser confocal microscopy, and the positively stained cells were counted. The proportion of positive cells observed under by laser confocal microscopy was counted, and statistical analysis was performed using the Student’s t test.

### Animal experiments

All animal experiments were approved by the Medical Ethics Committee of Tongji Medical College of Huazhong University of Science and Technology and conducted under the guidance of the World Declaration on Animal Welfare. Four- to six-week-old BALB/c-nu female mice were randomly grouped and blindly fed, the conditions of the mice are consistent between groups except for the experimental factors. Stable control and REV1 knockdown lung cancer cells were diluted to 1 × 10^7^ cells/mL in serum-free medium and each mouse was inoculated with 100 µL of cell suspension. Mice requiring radiotherapy were treated with 10 Gy x 1 F radiation treatment (X-ray irradiator, Varian, USA, dose rate 6 Gy/min). For JH-RE-06 treatment, JH-RE-06 was dissolved in a solvent containing 10% EtOH, 40% PEG400, and 50% saline and 1.6 mg/kg was administered by intraperitoneal injection twice a week until the tumor volume reached approximately 100 mm^3^ [6]. Mice in the combined drug and radiotherapy group were treated with radiotherapy the day after drug treatment. The growth and weight curves were plotted after observing and recording the long and short tumor diameters and weighing the mice every 3 days. Tumor doubling time (TDT) was measured based on the tumor growth curves. Enhancement factor (EF) of JH-RE-06 was calculated according to the formula “EF = $\frac{\text{T}\text{D}\text{T} \left(\text{J}\text{H}-\text{R}\text{E}-06 +\text{I}\text{R}\right) - \text{T}\text{D}\text{T} (\text{J}\text{H}-\text{R}\text{E}-06)}{\text{T}\text{D}\text{T} \left(\text{V}\text{e}\text{h}\text{i}\text{c}\text{l}\text{e}+\text{I}\text{R}\right) - \text{T}\text{D}\text{T} \left(\text{V}\text{e}\text{h}\text{i}\text{c}\text{l}\text{e}\right)}$”. EF > 1 indicates a radiosensitizing effect of JH-RE-06 [24–26].

### Liquid chromatography-tandem mass spectrometry (LC-MS/MS)

LC-MS/MS is mainly used to detect the metabolite content of tissues, serum, cells, and cell supernatants. First, sample metabolites were extracted by obtaining each group of experimental samples (tissue, serum, cell, and cell supernatant), extracting the metabolites according to the experimental requirements, diluting the metabolites and then storing the samples at 4 ℃. Then, the standard solution was prepared by accurately weighing the corresponding amount of standards in a 10 mL volumetric flask and preparing 1 mmol/L standard stock solution. The required amount of standard stock solution was placed in a 10 mL volumetric flask, and a mixed standard solution was prepared. Then, UHPLC-MS detection was performed by subjecting the target compounds to chromatography on a Waters Xbridge Amide (2.1 mm × 100 mm, 3.5 μm) liquid chromatographic column using an Agilent 1290 Infinity II series (Agilent Technologies) ultrahigh-performance liquid chromatograph. The A-phase of the liquid chromatography was 0.1% formic acid in water, and the B-phase was acetonitrile. The mobile phase flow rate was 300 µL/min, the column temperature was 35 °C, the sample tray temperature was 10 °C, and the injection volume was 1 µL.

### Enzyme-linked immunosorbent assay (ELISA)

Cells were transfected with the corresponding siRNAs for 48 h and then replaced with serum-free RPMI-1640 without Gly/Ser/Thr. Cell supernatants and cell lysates were collected, and the levels of Gly/Ser/Thr were determined using the corresponding ELISA kits (KT210400, BA21441, and BA21442, Moshak), respectively. Briefly, the standards and the samples were added to the enzyme-coated plate and incubated with the enzyme reagent at 37 ℃ for 1 h. After 5 times washing, the chromogenic solution was added and the plate was incubated at 37 ℃ for 15 min, protected from light. The absorbance at 450 nm was measured after the addition of the termination solution. The standard curve was plotted, and the final concentrations of the samples were calculated according to the absorbance value and the dilution ratio.

### Statistical analysis

All experimental data were statistically analyzed using GraphPad Prism 8 software. If not otherwise specified, the comparison of differences between two groups of measurement data was performed by t test, and multiple comparisons of repeatedly measured tumor volume growth data were performed by one-way ANOVA. n.s. *P* > 0.05, *P < 0.05, **P < 0.01, ***P < 0.001.

## Results

### Targeting REV1 enhances lung cancer radiosensitivity in vivo but not in vitro

**Fig. 1:**
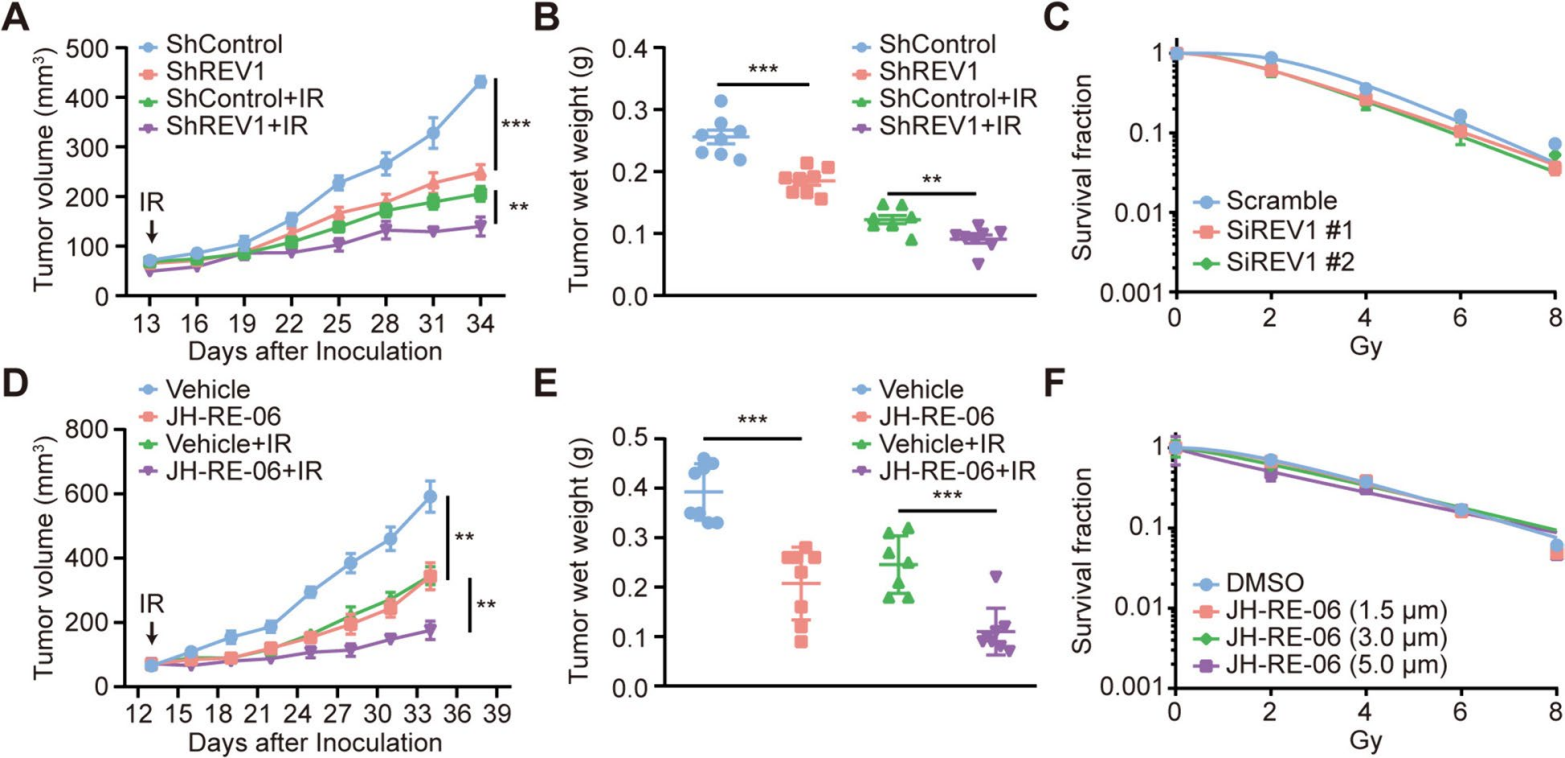
Targeting REV1 enhances lung cancer radiosensitivity in vivo but not in vitro*. ***A** Control or stable knockdown REV1 A549 lung cancer cells were selected to construct a subcutaneous transplantation tumor model in nude mice, which were then randomly divided into four groups. A total of 10 Gy x 1 F radiation treatment was given to the radiotherapy group after the tumor reached 100 mm^3,^ and the tumor volume was measured every three days. Data are shown as the mean tumor volume ± SEM. **P < 0.01, ***P < 0.001 (*n* = 8 or 7 mice/group). **B** Weight of transplanted tumors in mice of each group in panel **A**. **P < 0.01, ***P < 0.001 (*n* = 8 or 7/group). **C** A549 cells were transfected with control or siREV1 for 48 h, digested, and counted. Cells were irradiated with 0, 2, 4, 6, and 8 Gy X-rays after wall attachment, and the number of cell groupings with more than 50 cells were considered colonies and was recorded after 14 days of culture to obtain the cell survival curve. **D** A549 lung cancer cells were used to generate a subcutaneous transplantation tumor model in nude mice and mice were randomly divided into four groups. JH-RE-06 was used at a final concentration of 1.6 mg/kg, and the drug was administered by intraperitoneal injection twice a week beginning once the tumor volume reached 100 mm^3^. The experimental group requiring radiotherapy was subjected to 10 Gy of radiotherapy 1 day after drug administration, and tumor volume was measured every three days. Data are shown as the mean tumor volume ± SEM. **P < 0.01 (*n* = 8 or 7 mice/group). The tumor doubling time (TDT) for Vehicle, JH-RE-06, Vehicle + IR, and JH-RE-06 + IR was 2.82 ± 0.58 days, 4.06 ± 1.13 days, 4.01 ± 0.30 days, and 5.98 ± 2.00 days, respectively. The enhancement factor (EF) of JH-RE-06 was 1.61. **E** Weight of transplanted tumors in each group of mice in the **D** graph. ***P < 0.001 (*n* = 8 or 7/group). **F** After treatment of A549 cells with different concentrations of JH-RE-06 for 24 h, digestions were counted on spread plates, and cells were irradiated with corresponding doses of X-ray after wall attachment. After 14 days, the number of cell spheres with more than 50 cells was recorded, and cell survival curves were depicted

To investigate the role of REV1 in lung cancer radiotherapy, we performed radiosensitivity assays in vivo and in vitro. First, stable knockdown of REV1 expression in the A549 lung cancer cell line was achieved using shRNA technology (Figure S1A-B), and a subcutaneous transplantation tumor model was constructed in nude mice. After the average volume of transplanted tumors reached 100 mm^3^, mice were treated with radiation. The results showed that both REV1 knockdown and radiation treatment could inhibit the tumor growth rate and reduce the weight of transplanted tumors to a certain extent; the inhibition effect was most obvious in the combined treatment group, indicating that REV1 knockdown and radiation treatment had a synergistic effect (Fig. 1A-B, Figure S2A-B). Surprisingly, the results of in vitro experiments did not support this conclusion, as the knockdown of REV1 in A549 cells in vitro did not cause an increase in radiosensitivity (Fig. 1C, Figure S1C-D). To further confirm this discrepant result, we selected JH-RE-06, a functional inhibitor of REV1, and repeated the radiosensitivity assay. As shown in Fig. 1D-E and Figure S2C-D, JH-RE-06 also synergized with radiotherapy in vivo, while in vitro, JH-RE-06 with graded increasing concentrations selected according to its IC50 did not affect the radiosensitivity of lung cancer cells in vivo (Fig. 1F) [6]. These experiments suggest that targeting REV1 can enhance the radiosensitivity of lung cancer cells in vivo, but this effect is not observed in vitro.

### Targeting REV1 participates in the regulation of radiosensitivity of lung cancer by down-regulating the Gly/Ser/Thr metabolism

**Fig. 2:**
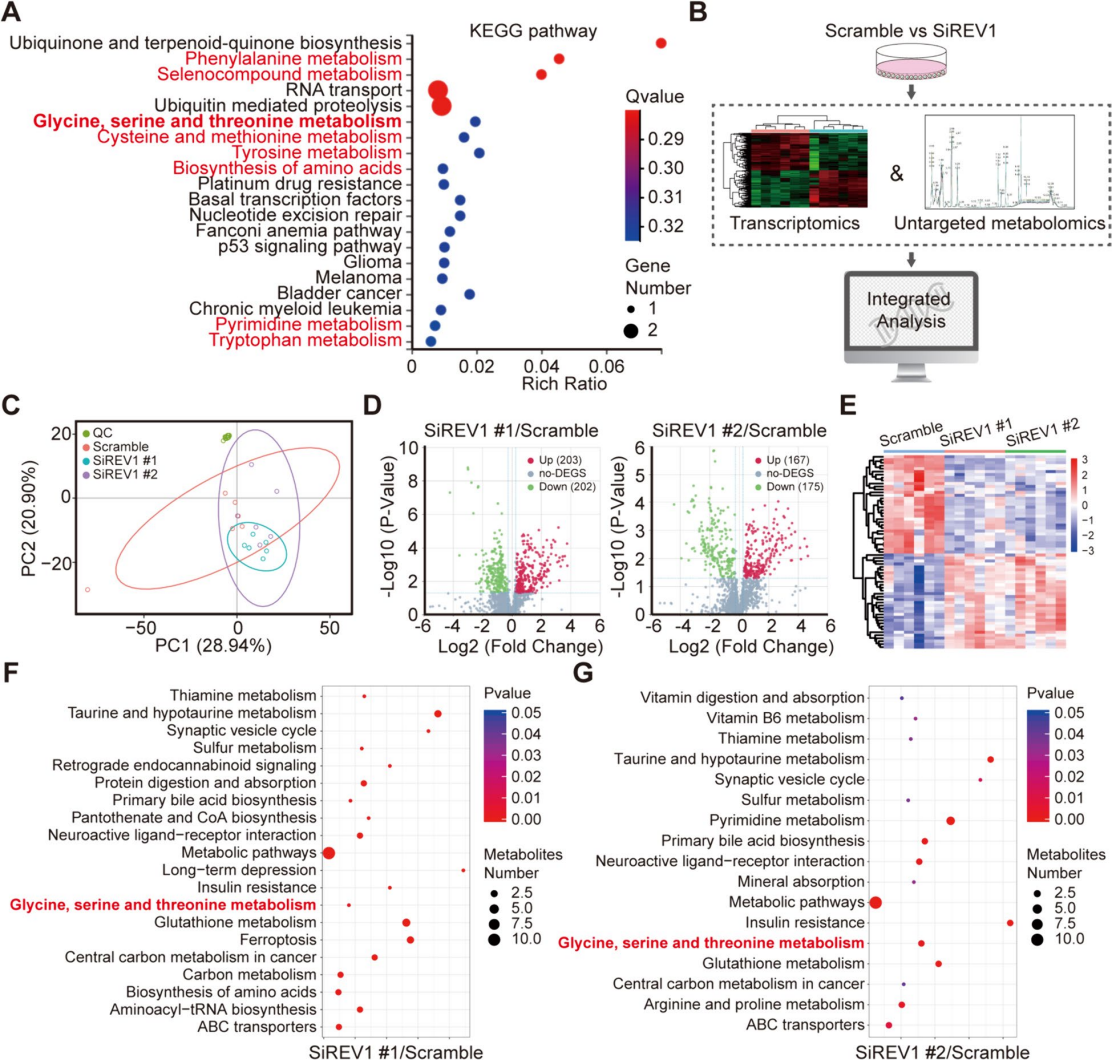
Targeting REV1 downregulates Gly/Ser/Thr signaling pathways. **A** RNA-seq after knockdown of REV1 by two different siRNA sequences, and the intersection of differentially expressed genes was taken for signaling pathway enrichment analysis and plotted in bubble plots. **B** Pattern plot of combined transcriptome and metabolome sequencing. **C** Plot of principal component analysis of nontargeted metabolic sequencing samples. **D** Volcano plot of differential metabolites (fold change absolute value ≥ 2, *P* < 0.05). **E** Heatmap of differential metabolite clustering. **F-G** Bubble map of differential metabolite pathway enrichment

To investigate the mechanism through which REV1-induced resistance to lung cancer radiotherapy occurs and the intrinsic reasons for the different effects of REV1 targeting on lung cancer radiosensitivity in vivo and in vitro, we identified the genes that were differentially expressed in the experimental and control groups and performed RNA-sequence assays and KEGG signaling pathway enrichment analysis. These analyses revealed that, in lung cancer cells after REV1 deletion, numerous metabolism-related pathways underwent significant enrichment, such as phenylalanine metabolism, selenocompound metabolism, and Gly/Ser/Thr metabolism, suggesting that the function of REV1 might be related to metabolic factors (Fig. 2A). To further clarify the specific metabolic mechanisms that function downstream of REV1, we next performed nontargeted metabolomics assays and combined the transcriptomic and metabolomic data (Fig. 2B). In the nontargeted metabolomics data, common differential metabolites identified after treatment with two different siRNA sequences were further analyzed for KEGG pathway enrichment, and the results are shown in Fig. 2C-G. The Gly/Ser/Thr metabolic pathways attracted our attention. We observed that both of the siRNAs caused the downstream enrichment of this pathway, and more importantly, transcriptomic analysis of the pathway also showed that it was enriched. Therefore, we speculate that the function of REV1 may be related to the Gly/Ser/Thr metabolic signaling pathways.

**Fig. 3:**
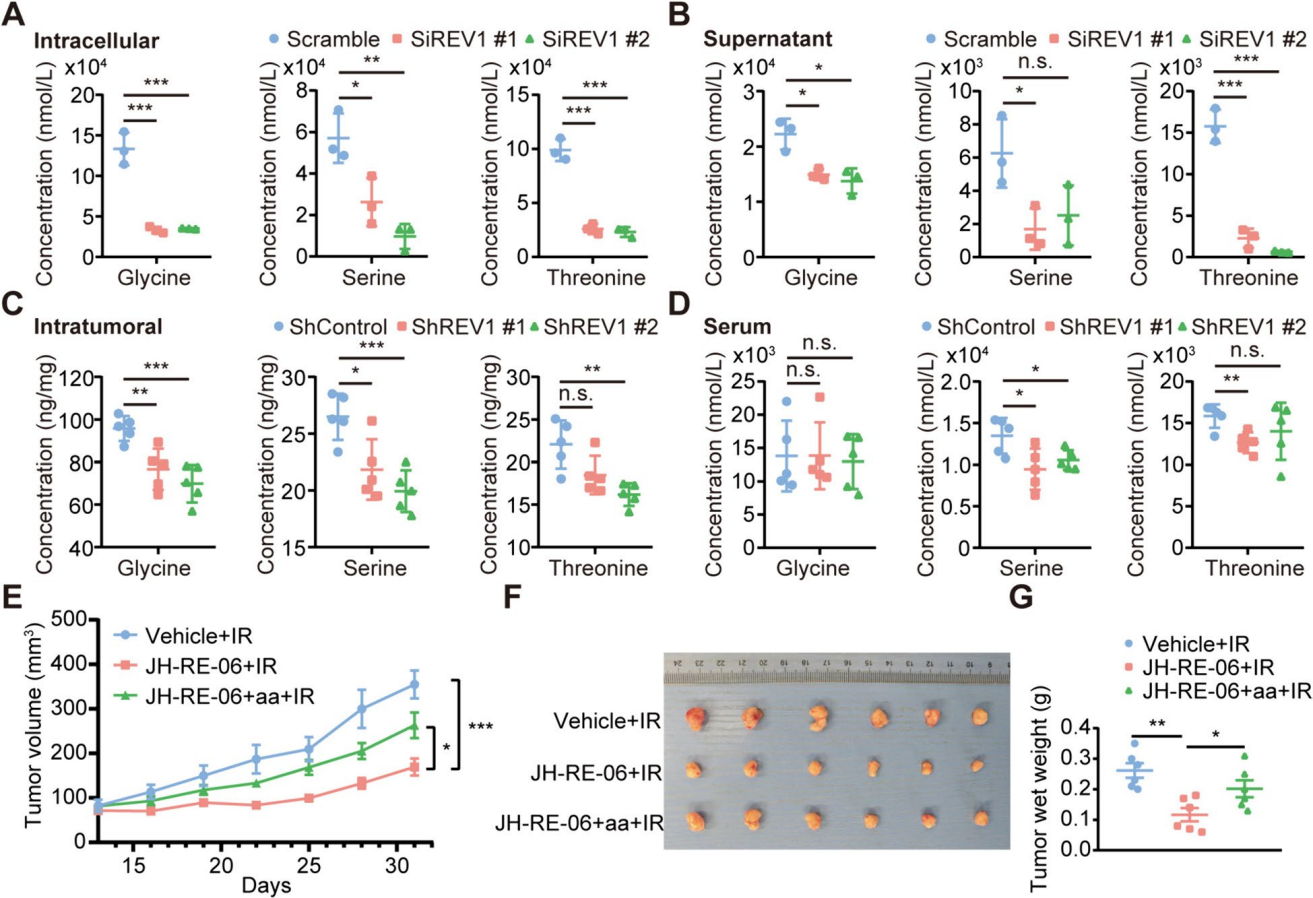
REV1 participates in the regulation of radiosensitivity in a manner dependent on the Gly/Ser/Thr metabolism. **A-D** LC-MS to the levels of Gly/Ser/Thr, intracellular (**A**), supracellular (**B**), intratumoral (**C**), and serum (**D**). *P < 0.05, **P < 0.01, (*n* = 3). **E** A subcutaneous transplantation tumor model was constructed, and drug administration was started intraperitoneally twice a week after the tumor had grown to the target volume. Each group was given 10 Gy of 1 radiation treatment 1 day after dosing. The mice in the experimental group were given an amino acid mixture by gavage (Gly/Ser/Thr isometric mixture, 500 mg/kg/d), and the remaining experimental groups were given equivalent placebo treatment. Tumor volumes were measured every three days. Data are shown as the mean tumor volume ± SEM. **P < 0.01 (*n* = 6 mice/group). The tumor doubling time (TDT) for Vehicle + IR, JH-RE-06 + IR, and JH-RE-06 + aa + IR was 3.37 ± 0.65 days, 5.83 ± 0.85 days, and 3.90 ± 0.67 days, respectively. **F** The tumor was surgically removed and photographed at the end of the experiment. **G** The weight of transplanted tumors in each group of mice in panel **B**. *P < 0.05, **P < 0.01 (*n* = 6 mice/group)

Validation using liquid chromatography-tandem mass spectrometry revealed that REV1 knockdown resulted in different levels of Gly/Ser/Thr expression downregulation both intra- and extracellularly and within tumors. In contrast, for amino acids in serum, no significantly different and consistent alterations in Gly/Ser/Thr levels were observed; however, serine levels were significantly altered (Fig. 3A-D). The above experiments suggest that targeting REV1 can downregulate the expression levels of these three amino acids both intra- and extracellularly and in tumors while having little effect on the serum levels. In addition, radiotherapy itself does not influence the content of the specific amino acids (Figure S3), indicating that REV1 inhibition-induced radiosensitization in vivo may be attributed to reduced amino acid levels resulting from targeting REV1. To confirm this conjecture, we performed an in vivo rescue experiment. We exogenously supplemented mice receiving JH-RE-06 in combination with radiotherapy with amino acids and found that the exogenous addition of amino acids partially attenuated the radiosensitizing effect of JH-RE-06, suggesting that amino acid metabolism is the key to REV1-induced radioresistance (Fig. 3E-G).

#### REV1 downregulates CTH expression in an E3 ubiquitin ligase Rad18-dependent manner

**Fig. 4:**
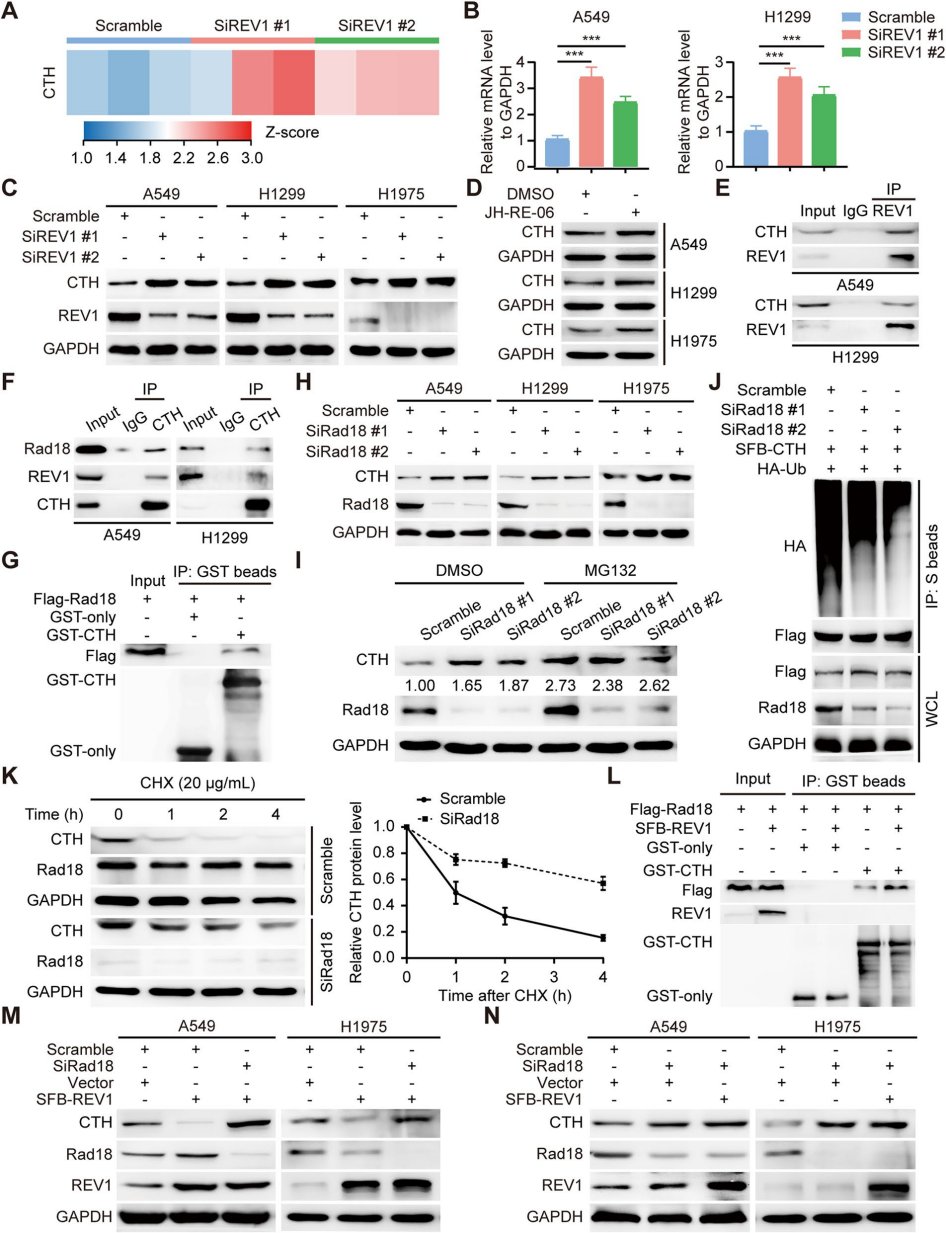
REV1 downregulates CTH expression by promoting Rad18 binding and ubiquitinating CTH for degradation. **A** Heatmap generated from the results of enrichment of Gly/Ser/Thr signaling pathways in RNA sequencing. **B** REV1 was knocked down in A549 and H1299 cells, and the relative mRNA levels of CTH were detected by RT-PCR. ***P < 0.001, (*n* = 3). **C** A549, H1299, and H1975 cells were transfected with siREV1, and the expression of the corresponding antibody was detected by Western blotting after 48 h (*n* = 3). **D** DMSO and JH-RE-06-treated A549, H1299, and H1975 cells were harvested and CTH expression was detected by Western blotting (*n* = 3). **E-F** Endogenous interaction of CTH with REV1 and Rad18 in A549 cells and H1299 cells. **G** Proteins of HEK293T cells transfected with Flag-Rad18 were collected and added to GST-only and GST-CTH fusion proteins, respectively. The expression of the corresponding molecules was detected by Western blotting after overnight enrichment and elution (*n* = 3). **H** A549, H1299, and H1975 cells were transfected with siRad18, and the expression of the corresponding proteins was detected by Western blotting after 48 h (*n* = 3). **I** Scrambled and Rad18-deleted A549 cells were treated with DMSO or MG132 for 4 h before protein collection, and the expression of the indicating molecules was examined by Western blotting (*n* = 3). **J** Cells were transfected with the corresponding plasmids and siRNA, MG132 was added 4 h before protein collection treatment, and the in vitro CTH ubiquitination level was detected using the immunoprecipitation technique (*n* = 3). **K** Left panel: Scrambled and Rad18-deleted A549 cells were treated with CHX (20 µg/mL). Cellular proteins were collected at the corresponding times, and the protein levels of CTH were detected by Western blotting (*n* = 3). Right panel: quantitative statistical analysis of CTH protein (*n* = 3). **L** Proteins of HEK-293T cells transfected with the corresponding plasmids were harvested and incubated with GST-only or GST-CTH fusion proteins overnight and analyzed by Western blotting (*n* = 3). **M-N** A549 and H1975 cells were transfected with the corresponding siRNA or plasmid and analyzed by Western blotting using the corresponding antibodies (*n* = 3)

To further investigate the specific mechanism by which REV1 regulates this altered metabolic signaling pathway, we analyzed the transcriptomic data and found that at the transcriptional level, the Gly/Ser/Thr metabolic signaling pathway was enriched for one gene, CTH (Fig. 4A), which encodes a cytoplasmic enzyme involved in Gly/Ser/Thr catabolism. This gene encodes a cytoplasmic enzyme involved in the catabolism of Gly/Ser/Thr through mediating the conversion of methionine-derived cystathionine to cysteine, and CTH expression can downregulate the intra- and extracellular levels of Gly/Ser/Thr [27]. Targeted inhibition of REV1 was found to upregulate CTH expression as observed by both RT-PCR and Western blotting results (Fig. 4B-D). The above experiments suggest that REV1 has a negative regulatory effect on CTH. It has been reported that REV1 can act as a scaffolding protein to promote the ubiquitination degradation of a substrate by binding of the E3 ligase Rad18 to substrate molecules [28, 29], so does REV1 also downregulate CTH expression through this mechanism? To investigate this, our co-IP experiments first found that CTH interacted with REV1 and Rad18, which is further confirmed by the GST pull-down assay (Fig. 4E-G). Then does the regulatory effect of Rad18 on CTH depend on its classical E3 function? Upregulation of CTH expression was detected after treatment with the proteasome inhibitor MG132, suggesting that CTH is regulated by the ubiquitin-proteasome system (Figure S4). Knockdown of Rad18 in three different lung cancer cells resulted in CTH upregulation, and treatment with MG132 significantly inhibited this observation (Fig. 4H-I). In addition, silencing Rad18 downregulated the ubiquitination level of CTH and prolonged its protein half-life (Fig. 4J-K). Importantly, overexpression of REV1 increased the protein interaction between CTH and Rad18, indicating that REV1 can serve as a molecular scaffold to facilitate Rad18 binding to its substrate CTH (Fig. 4L). To verify this mechanism, we conducted a rescue experiment to determine whether Rad18 is involved in the regulation of CTH by REV1. We first exogenously overexpressed REV1 in lung cancer cells and detected the downregulation of CTH expression. In cells simultaneously knocking down Rad18 and overexpressing REV1, CTH expression was significantly upregulated. In addition, we detected upregulation of CTH expression upon knockdown of Rad18, while simultaneous REV1 overexpression and Rad18 knockdown did not change CTH expression compared to knockdown of Rad18 alone (Fig. 4M-N). The above results suggest that REV1 can negatively regulate CTH expression, and that this process is dependent on the E3 ligase Rad18.

### Targeting REV1 enhances lung cancer radiosensitivity by inducing alterations in the Gly/Ser/Thr metabolism dependent on CTH

**Fig. 5:**
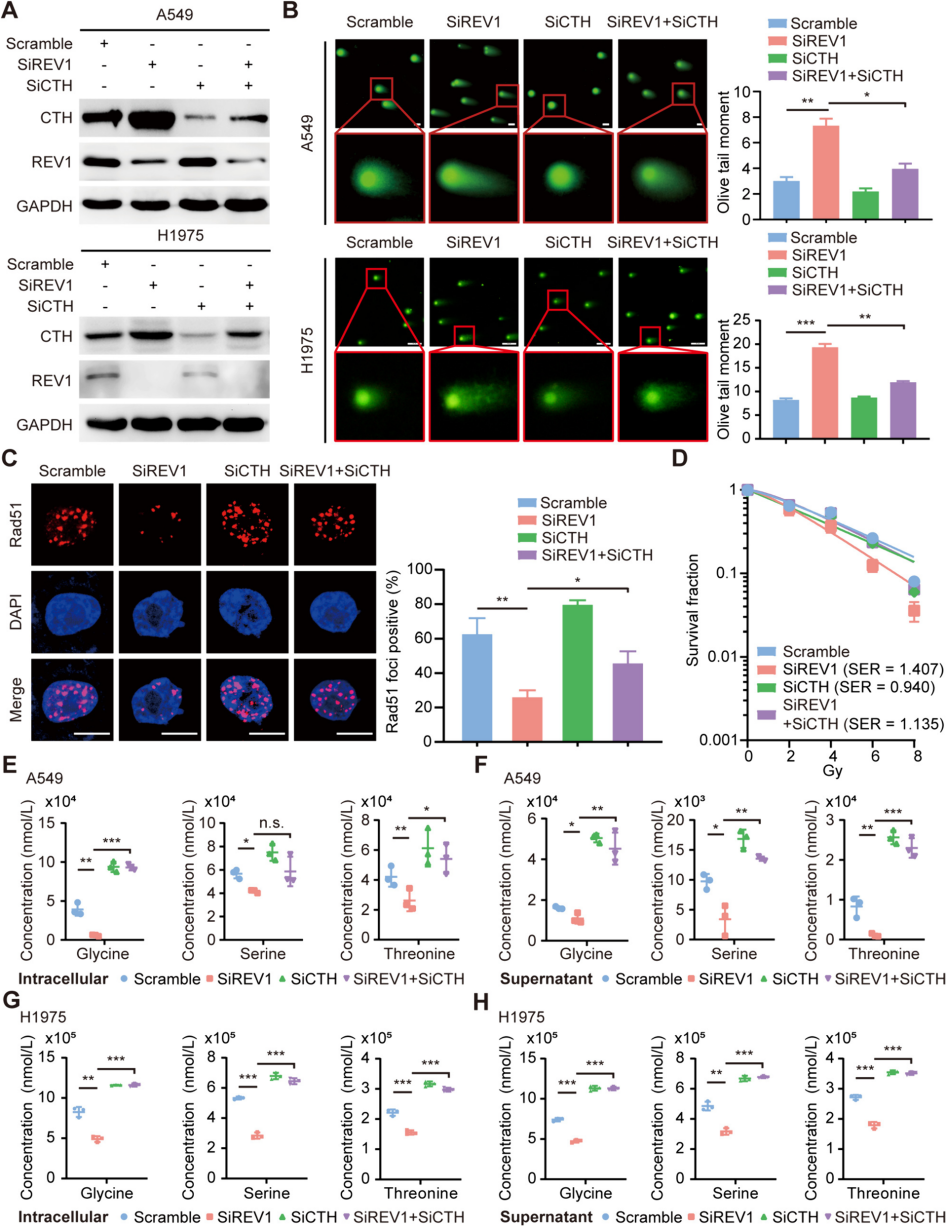
Targeted REV1 enhances lung cancer radiosensitivity by inducing alterations in the Gly/Ser/Thr metabolism dependent on CTH. **A** A549 and H1975 cells were transfected with the corresponding siRNA, and the expression of the corresponding protein was detected using Western blotting (*n* = 3). **B** Representative images of A549 and H1975 cells receiving the corresponding treatment and IR for 4 h followed by comet trailing assay. Bar, 20 μm. **C** Cells in each group received the corresponding treatment, focal staining was performed with Rad51 antibody, and the proportion of focal positive cells was counted using fluorescence microscopy. Bar, 10 μm. **D** Cells in each group received the corresponding dose of irradiation after treatment, and the number of colonies containing more than 50 cells was counted after continued incubation for 2 weeks (*n* = 3). **E-H** Intracellular and cell supernatant Gly/Ser/Thr levels in A549 and H1975 cells. n.s. *P* > 0.05, *P < 0.05, **P < 0.01, ***P < 0.001 (*n* = 3)

To confirm the role of CTH in REV1-mediated remodeling of the metabolic microenvironment, a series of rescue experiments were conducted using a custom conditioned medium (RPMI-1640 without Gly/Ser/Thr). The findings showed that REV1 silencing resulted in a prolonged comet tail, a reduced number of Rad51 foci, and a decreased colony formation ability, indicating an increased cell radiosensitivity (Fig. 5A-D). Consistently, overexpression of REV1 exhibited impaired sensitivity to irradiation (Figure S5). What’s more, REV1 silencing-mediated radiosensitization could be reversed by CTH knockdown, suggesting that REV1 induces radioresistance in a CTH-dependent manner (Fig. 5A-D). Importantly, the levels of the abovementioned three amino acids were significantly decreased after REV1 knockdown, both intracellularly and in the cell supernatant, while simultaneous knockdown of CTH and REV1 partially restored the levels of the three amino acids (Fig. 5E-H). In conclusion, our results suggest that REV1 induces radioresistance by affecting the Gly/Ser/Thr metabolism, and that this process is dependent on the expression of CTH.

### USP9X positively regulates REV1 expression through the ubiquitin-proteasome system pathway

**Fig. 6:**
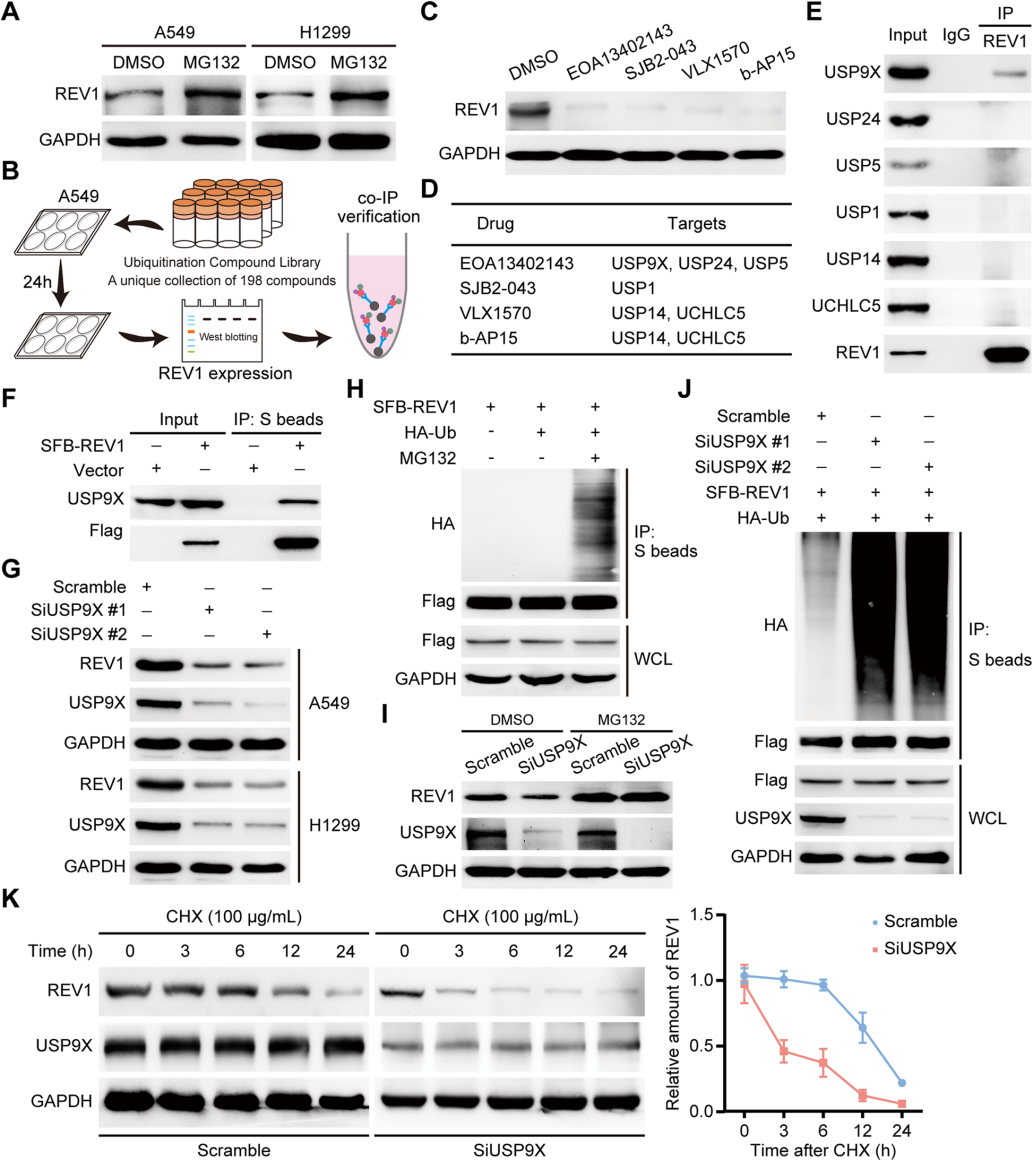
USP9X positively regulates REV1 expression through the ubiquitin-proteasome system pathway. **A** Protein extracted from A549 and H1299 lung cancer cells after 4 h of MG132 treatment and protein expression of REV1 detected by Western blotting. **B** Flow chart of the ubiquitination compound library screening system. **C** The four drugs obtained from the screening that inhibited REV1 protein expression most significantly were used to treat A549 cells for 24 h. The proteins were extracted, and the expression of REV1 protein was detected by Western blotting. **D** The targets of the four small-molecule drugs obtained from the screening. Interaction between USP9X and REV1 (*n* = 3). **E** Endogenous interaction between REV1 and small molecule drug targets. **F** A549 cells were transfected with SFB-REV1 plasmid, and the interaction between USP9X and REV1 was detected by co-IP assay after 24 h (*n* = 3). **G** siUSP9X was transfected into A549 and H1299 lung cancer cells, and REV1 expression was detected by Western blotting after 48 h (*n* = 3). **H** A549 cells were transfected with the corresponding plasmids for 24 h, treated with MG132 for 4 h before protein extraction, and treated with S beads for immunoprecipitation. **I** A549 cells were transfected with Scramble or siUSP9X for 48 h, DMSO or MG132 was added 4 h before protein extraction, and the expression of the corresponding proteins was detected by Western blotting (*n* = 3). **J** Cells were transfected with the corresponding siRNA or plasmid, MG132 was added 4 h before protein extraction, and immunoprecipitation experiments were performed with S beads for immunoprecipitation experiments (*n* = 3). **K** Left panel: A549 lung cancer cells transfected with scramble or siUSP9X for 48 h before CHX (100 µg/mL) treatment. Cellular proteins were collected at the corresponding times, and the protein levels of REV1 were detected by Western blotting (*n* = 3). Right panel: quantitative statistical analysis of REV1 protein (*n* = 3)

More than 80% of eukaryotic proteins are regulated by the ubiquitin-proteasome system. To identify the specific molecules that regulate REV1, we first treated lung cancer cells with MG132 and found that REV1 expression was significantly upregulated, suggesting that REV1 may be regulated by the ubiquitin-proteasome system (Fig. 6A). Subsequently, we constructed a ubiquitination compound library screening system containing 198 small molecule drugs. Using this library, we screened four drugs that most significantly inhibited REV1 protein expression, and performed REV1 co-IP analysis on the targets of these drugs. This analysis which showed that USP9X interacted with REV1 (Fig. 6B-E). Similarly, exogenously expressed REV1 was able to form a complex with intracellularly expressed USP9X (Fig. 6F). Furthermore, knockdown of USP9X in two different lung cancer cell lines resulted in the downregulation of REV1 expression, suggesting positive regulation of REV1 by USP9X (Fig. 6G). Mechanistically, USP9X, as a deubiquitinase, can stabilize its expression by affecting the ubiquitination level of the substrate molecule. Ubiquitination assay results indicated REV1 can be significantly ubiquitinated in lung cancer cells, and the positive regulatory effect of USP9X on REV1 can be reversed by MG132 (Fig. 6H-I). Importantly, knockdown of USP9X enhanced the ubiquitination level of REV1 and significantly shortened REV1 protein half-life (Fig. 6J-K). The above results suggest that the deubiquitinase USP9X can stabilize REV1 expression in lung cancer cells by mediating its deubiquitination modification.

### USP9X reshapes the amino acid metabolic microenvironment by stabilizing REV1 expression, thereby promoting resistance to lung cancer radiotherapy

**Fig. 7:**
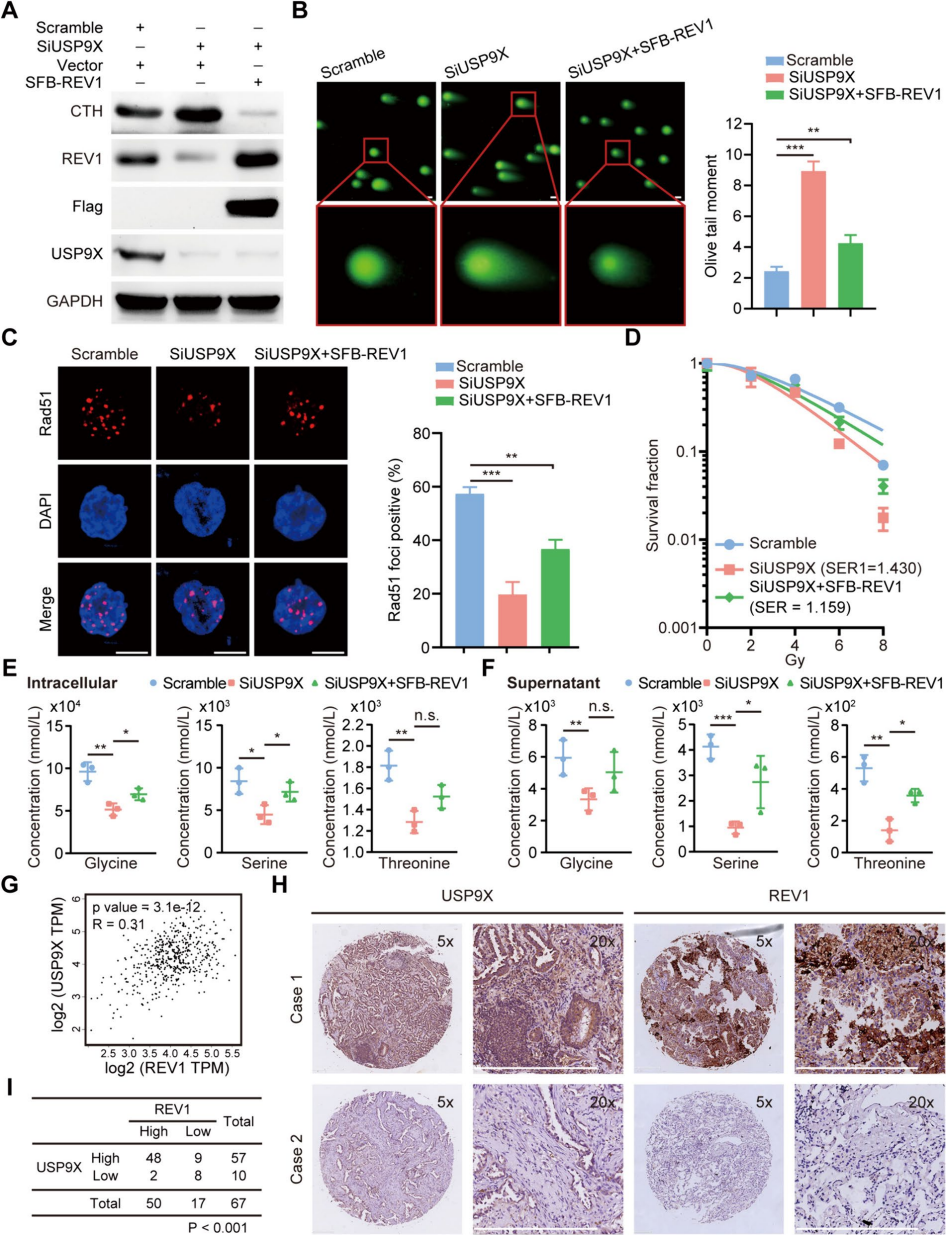
USP9X reshapes the amino acid metabolic microenvironment by stabilizing REV1 expression, thereby promoting lung cancer radioresistance. **A** A549 cells were transfected with siRNA or SFB-REV1 plasmids, and Western blotting was used to detect the expression levels of the corresponding proteins (*n* = 3). **B** Left panel: typical representative images of the comet assay; scale bar, 10 μm. Right panel: Olive tail moment statistics, **P < 0.01, ***P < 0.001 (*n* = 3). **C** Left panel: A549 cells transfected with SFB-REV1 and/or siUSP9X  4 h after receiving 6 Gy of 1 irradiation and immunostaining to detect Rad51 focus formation. Right panel: statistical analysis of the proportion of Rad51 focus-positive cells, **P < 0.01, ***P < 0.001 (*n* = 3). **D** A549 cells transfected with SFB-REV1 and/or SiUSP9X, 2 weeks after receiving the corresponding dose of X-ray irradiation, number of dose clones, and plotted cell survival curves. **E** LC-MS to detect intracellular levels of Gly/Ser/Thr. *P < 0.05, **P < 0.01, (*n* = 3). **F** LC-MS to detect cell supernatant Gly/Ser/Thr levels. n.s. *P* > 0.05, *P < 0.05, **P < 0.01, ***P < 0.001 (*n* = 3). **G** The data from GEPIA showed that the expression of USP9X was positively correlated with that of REV1. **H** Representative immunohistochemical staining for USP9X and REV1 in lung cancer tissues. Scale bar, 400 μm. **I** Positive correlation between USP9X and REV1 protein levels in lung cancer tissues (*P* < 0.001, chi-square test)

To further clarify the role of USP9X-stabilized REV1 expression in lung cancer radioresistance and to elucidate whether this process is associated with alterations in the metabolic microenvironment, we transferred exogenously expressed REV1 into USP9X-deficient lung cancer cells. We found that REV1 expression was downregulated when USP9X was knocked down, while CTH expression was significantly upregulated. Importantly, overexpression of REV1 in USP9X-deficient lung cancer cells was able to reverse the upregulation of CTH expression (Fig. 7A). Additional functional experiments revealed that USP9X-deficient lung cancer cells formed longer comet trails, fewer Rad51 foci, and exhibited enhanced radiosensitivity after radiation exposure. These phenotypes were partially reverted by exogenous overexpression of REV1, suggesting that USP9X could induce radioresistance in lung cancer cells by stabilizing REV1 expression (Fig. 7B-D). More importantly, we examined the intra- and extracellular levels of three amino acids, and as expected, knockdown of USP9X downregulated the expression of these specific amino acids, while REV1 overexpression also partially reversed this phenotype (Fig. 7E-F). These results suggest that USP9X induces remodeling of the amino acid metabolic microenvironment by stabilizing the expression of REV1, thus promoting radiation resistance in lung cancer cells.

## Discussion

In this study, through a combined transcriptomic and nontargeted metabolomic approach, we found that the initial differential effects of REV1 on lung cancer radiosensitivity in vitro and in vivo were caused by differences in the metabolic microenvironment. Mechanistically, the ubiquitination compound library screening system identified the USP9X deubiquitinase as a novel upstream REV1 regulatory protein, and that USP9X mediated REV1 deubiquitination modifications to stabilize its expression in lung cancer. Aberrantly expressed REV1 acts as a scaffolding protein to assist the interaction between the Rad18 E3 ubiquitin ligase and CTH, promoting CTH ubiquitination-mediated degradation and inducing remodeling of the amino acid metabolic microenvironment, leading to lung cancer radioresistance. In addition, JH-RE-06, a novel small-molecule inhibitor of REV1, was shown to enhance lung cancer cell radiosensitivity in vitro and in vivo, with good clinical translation prospects.

REV1 is thought to be an important signaling node linking nutrient sensing and metabolic control to cell fate, and altered cellular metabolism is one of the hallmarks of tumorigenesis [30–32]. Sander Kooijman et al. found that REV1 deficiency leads to increased oxidative stress and mitochondrial dysfunction due to rapid NAD^+^ depletion by polyribose polymerase 1 (PARP1). There is an interaction between ineffective DNA damage repair and metabolic imbalance, which in turn is an important factor affecting tumor radiosensitivity; therefore, REV1 is expected to be a therapeutic target for enhancing tumor radiosensitivity [30]. However, a study by Nimrat Chatterjee et al. found that inhibition of REV1 failed to sensitize cancer cells to ionizing radiation, which is possibly related to the induction of autophagy, and suggested that REV1 inhibition is not a feasible synergistic approach for radiation treatment of cancer cells [33]. This is consistent with our initial in vitro results; however, we observed that REV1 inhibition acts synergistically with radiotherapy in vivo. In investigating the intrinsic reason for this result, we performed transcriptome sequencing and found that REV1 is closely related to metabolic factors, and further analysis using nontargeted metabolomics sequencing revealed that the Gly/Ser/Thr metabolic signaling pathways are keys to its function. This explains the discrepancy between our in vivo and in vitro results, as we used complete media with sufficient amounts of all amino acids for our in vitro experiments, ignoring the important effect of amino acids themselves on radiosensitivity. The abnormal expression of REV1 in tumor cells was found to activate the Gly/Ser/Thr metabolic signaling pathways, and the levels of Gly/Ser/Thr were significantly elevated both intra- and extracellularly. The abnormal metabolism of these three amino acids is an important factor in inducing tumor resistance to radiotherapy. Next, we performed in vitro experiments using conditioned media with depletion of specific amino acids and found that after radiation treatment, REV1-deficient lung cancer cells exhibited extended comet tails, a reduced number of Rad51 foci, and increased radiosensitivity. In addition, we performed in vivo rescue experiments and found that the exogenous addition of amino acids partially attenuated the radiosensitizing effect of JH-RE-06. Combining the results of in vitro and in vivo experiments, it can be concluded that REV1 inhibition enhances the radiosensitivity of lung cancer cells and that this process is associated with Gly/Ser/Thr metabolism.

Among the many TLS polymerases, REV1 has unique properties, and in addition to its unique catalytic activity, REV1 also has important noncatalytic functions [34, 35]. Some researchers found that REV1 can act as a scaffolding protein to facilitate the binding of the E3 ligase Rad18 to the substrate molecule for degradation. By targeting different substrate molecules, Rad18 induces radioresistance formation in various tumors such as glioma, hepatocellular carcinoma, and esophageal carcinoma, suggesting that Rad18 may be a key mediator in REV1 regulating downstream gene expression and exerting function [28, 29, 36, 37]. In our study, by integrated analysis of transcriptomic and nontargeted metabolomic sequencing data, we identified CTH, a regulator of REV1 regulation of Gly/Ser/Thr metabolism, a cytoplasmic enzyme that participates in Gly/Ser/Thr catabolism by mediating the conversion of methionine-derived cystathionine to cysteine, thereby downregulating intra- and extracellular Gly/Ser/Thr levels [27]. Based on the scaffolding protein role played by REV1 during Rad18 binding to substrates, we hypothesized that REV1 also negatively regulates CTH expression in a Rad18-dependent manner. To test this conjecture, we first confirmed that CTH interacts with REV1 and Rad18 by immunoprecipitation and GST pull-down assays. The expression of CTH was upregulated after knockdown of Rad18 in lung cancer cells, and Rad18 silencing resulted in reduced ubiquitination levels and prolonged protein half-life of CTH, suggesting that CTH is a substrate for the E3 ubiquitin ligase Rad18. Importantly, the rescue assay revealed that CTH expression was downregulated in lung cancer cells overexpressing REV1. Simultaneously, after knockdown of Rad18, CTH expression was significantly upregulated. In addition, upregulation of CTH expression was detected upon knockdown of Rad18, while overexpression of REV1 along with knockdown of Rad18 did not alter CTH expression compared to Rad18 knockdown alone. The above results indicate that the negative regulatory effect of REV1 on CTH requires Rad18, and in the absence of Rad18, the regulatory effect of REV1 on CTH is not observed. Taken together, REV1 acts as a molecular scaffold to facilitate the binding between Rad18 and CTH, which in turn promotes the ubiquitin-mediated degradation of CTH and leads to CTH downregulation.

Ubiquitination is an important type of protein posttranslational modification (PTM) that plays a crucial role in controlling substrate degradation, and in eukaryotes, more than 80% of intracellular protein degradation is regulated by the ubiquitin-proteasome system (UPS) [38, 39]. A growing body of evidence suggests that key UPS enzymes are closely associated with human malignant tumorigenesis and play different biological functions in different types of tumors depending on the substrate. For example, in triple-negative breast cancer (TNBC), USP9X deubiquitinates and stabilizes PPP1R14B, leading to PPP1R14B overexpression in TNBC tissues, decreasing STMN1-mediated α-microtubule protein acetylation and microtubule stability, and promoting cell cycle progression, leading to paclitaxel resistance in TNBC cells [40]. In lung cancer, USP4 was identified as a negative regulator of TNFα-mediated lung cancer cell migration through deubiquitination of TRAF2 and TRAF6 [41]. Our previous study found that the deubiquitinase USP9X could interact with the histone lysine demethylase 4 C (KDM4C) to mediate KDM4C deubiquitination and thus upregulate its protein expression, and aberrantly expressed KDM4C upregulated the transcriptional expression of TGF-β2 by directly reducing the level of histone H3K9me3 in the promoter region of TGF-β2, which in turn activated the Smad/ATM/Chk2 signaling pathway, promoting DNA damage repair and leading to lung cancer radioresistance [19]. All members of the TLS polymerase Y family have a ubiquitin-binding domain, which is required for effective interaction with monoubiquitinated PCNA in response to DNA damage [42, 43]. However, the mechanism underlying the regulation of REV1 by the ubiquitin-proteasome system is not known. The upregulation of REV1 expression upon treatment with the broad-spectrum proteasome inhibitor MG132 suggests that REV1 is indeed regulated by the ubiquitin-proteasome system, and through a library screening system of ubiquitinated compounds, we identified USP9X as a possible deubiquitinase that functions as an upstream regulatory protein of REV1. Protein stability, in vitro ubiquitination level assays, and protein half-life experiments confirmed that USP9X can downregulate the ubiquitination of REV1, which positively regulates REV1 protein expression. To our knowledge, this is the first direct report that REV1 can be posttranslationally modified and deubiquitinated. Moreover, both the data from the GEPIA database and the detection of protein expression in lung cancer tissue microarray confirmed that the expression of USP9X was positively correlated with REV1 (Fig. 7G-I). Furthermore, we demonstrated that USP9X is involved in the development of radioresistance in a REV1-dependent manner and, importantly, that metabolic remodeling of specific amino acids plays a key role in this process. Thus, our study satisfactorily explains the aberrant expression of REV1 in lung cancer cells, i.e., overexpression of REV1, a newly identified USP9X substrate, is the result of USP9X-mediated deubiquitination.

JH-RE-06 binds to the evolutionarily conserved CTD domain (REV7 binding surface) of REV1, which acts as a “molecular glue” to “glue” the two REV1 proteins together, forming a nonfunctional dimer, thus preventing REV1-REV7 interaction and recruitment of POL ζ [44]. This novel TLS inhibitor has high affinity and high specificity compared to other TLS inhibitors that are active in vitro. It has been demonstrated that JH-RE-06 reduces cisplatin-induced mutagenesis, increases the sensitivity of tumor cells to cisplatin, almost completely inhibits tumor growth when combined with cisplatin, and significantly prolongs the survival time of hormonal mice [45]. Our study, on the other hand, suggests for the first time that JH-RE-06 can be used clinically as a sensitizer for lung cancer radiotherapy, and in our animal model, JH-RE-06 produced a significant inhibitory effect when used in combination with radiotherapy. Thus, our study provides a theoretical basis for the clinical translation of the REV1-targeted drug JH-RE-06 and presents a new idea for radiotherapy sensitization in patients with lung cancer.

## Conclusions

**Fig. 8:**
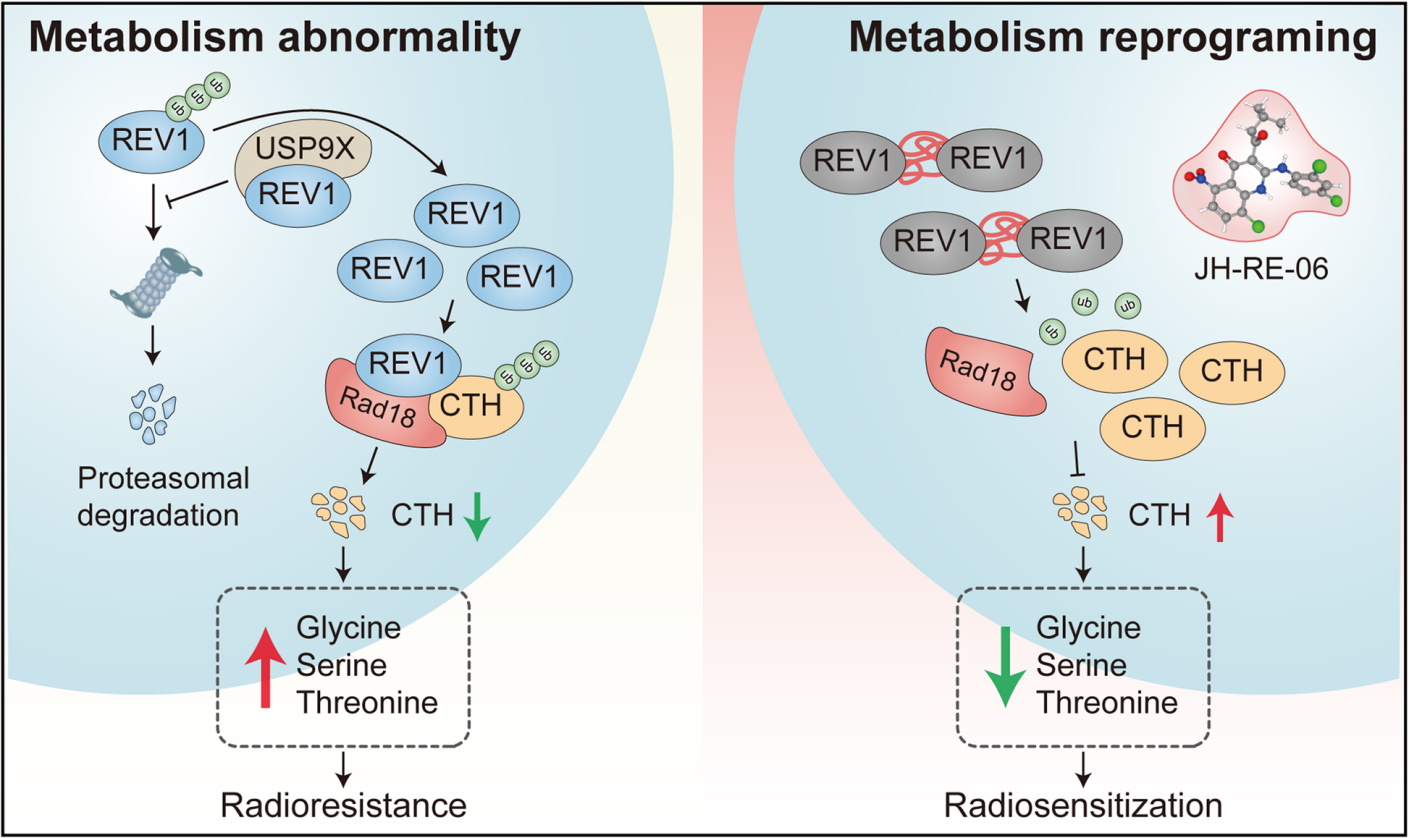
Schematic. Left: REV1 induces metabolic abnormalities involved in the development of radioresistance in lung cancer. USP9X mediates REV1 deubiquitination to stabilize its expression in lung cancer. Aberrantly expressed REV1 acts as a scaffolding protein to assist the E3 ubiquitin ligase Rad18 in interacting with CTH, promoting CTH ubiquitination degradation and upregulating intra- and extracellular Gly/Ser/Thr levels, leading to lung cancer radiotherapy resistance. Right: Therapeutic potential of targeting REV1 in lung cancer. JH-RE-06 promotes the formation of loss-of-function dimers of REV1 and induces remodeling of intra- and extracellular amino acid metabolism, thereby enhancing the radiosensitivity of lung cancer cells

In conclusion, our study revealed that REV1 plays an important role in the generation of radioresistance in lung cancer, that REV1 acts as a scaffolding protein to facilitate Rad18 binding to CTH, and that REV1 promotes CTH ubiquitination and degradation, thereby inducing the formation of an abnormal tumor metabolic microenvironment, which ultimately leads to the development of radioresistance. Furthermore, we revealed that the aberrant high expression of REV1 in lung cancer is caused by USP9X-mediated deubiquitination modifications. Importantly, we also provided preclinical evidence for clinical translation of REV1 targeting, demonstrating that JH-RE-06 can be used as a highly effective radiosensitizer for clinical applications (Fig. 8).

### Supplementary Information

Supplementary Material 1: Fig. S1. REV1 expression can be effectively knocked down by shRNAs and siRNAs. A The mRNA levels of the indicated molecules were measured by qRT-PCR in shControl and shREV1 lung cancer cells. *** *P* < 0.001 (*n* = 4). B REV1 knockdown cell line was successfully constructed by using shRNA technology (*n* = 3). C The mRNA levels of the indicated molecules were measured by qRT-PCR in scramble and siREV1 lung cancer cells.*** *P* < 0.001 (*n* = 4). D A549 cells transfected with the indicated siRNAs were harvested and analyzed by Western blotting (*n* = 3). 

Supplementary Material 2: Fig. S2. Targeting REV1 enhances the radiosensitivity of lung cancer cells *in vivo*. A REV1 was inhibited by shRNA knockdown and then combined with radiotherapy. Growth curves were plotted for each mouse (*n* = 8). B Photographs of transplanted tumors from the indicated groups (*n* = 8). C Growth curves for each mouse in JH-RE-06 combined with radiotherapy experiments were presented (*n* = 7). D Pictures of xenograft tumors from the corresponding groups (*n* = 7).

Supplementary Material 3: Fig. S3. Radiotherapy had little effect on glycine, serine, and threonine metabolism. The levels of glycine, serine, and threonine in the control and radiotherapy group were determined by LC-MS. n.s. *P* > 0.05 (*n* = 3).

Supplementary Material 4: Fig. S4. CTH is regulated by the ubiquitin-proteasome system. A549 cells (A) and H1299 cells (B) were treated with DMSO or MG132 for 4 h before protein extraction. The CTH expression was detected by Western blotting (*n* = 3).

Supplementary Material 5: Fig. S5. Overexpression of REV1 reduces lung cancer radiosensitivity *in vitro. *A A549 and H1299 cells were transfected with vector and SFB-REV1 and collected for Western blotting (*n* = 3). B A549 and H1299 cells transfected with vector and SFB-REV1 were subjected to comet assay after receiving 6 Gy irradiation, representative images and the statistics of comet tail moment are shown. *** *P* < 0.001 (*n* = 100). C A549 and H1299 cells transfected with vector and SFB-REV1 were subjected to Rad51 immunofluorescence staining after receiving 6 Gy irradiation. The proportion of Rad51 foci-positive cells was calculated under a fluorescence confocal microscope. ** *P* < 0.01, *** *P* < 0.001 (*n* = 3).

Supplementary Material 6: Table S1. Sequences of siRNA and shRNA used in this study. Table S2. Sequences of primers used for Real-time quantitative PCR.

## Data Availability

All data generated in the present study may be requested from the corresponding authors.
