# Supplementary figures and images for: USP9X-mediated REV1 deubiquitination promotes lung cancer radioresistance via the action of REV1 as a Rad18 molecular scaffold for cystathionine γ-lyase
Source: J Biomed Sci. 2024 May 28;31:55. doi: 10.1186/s12929-024-01044-3 (PMC11131313; doi:10.1186/s12929-024-01044-3)

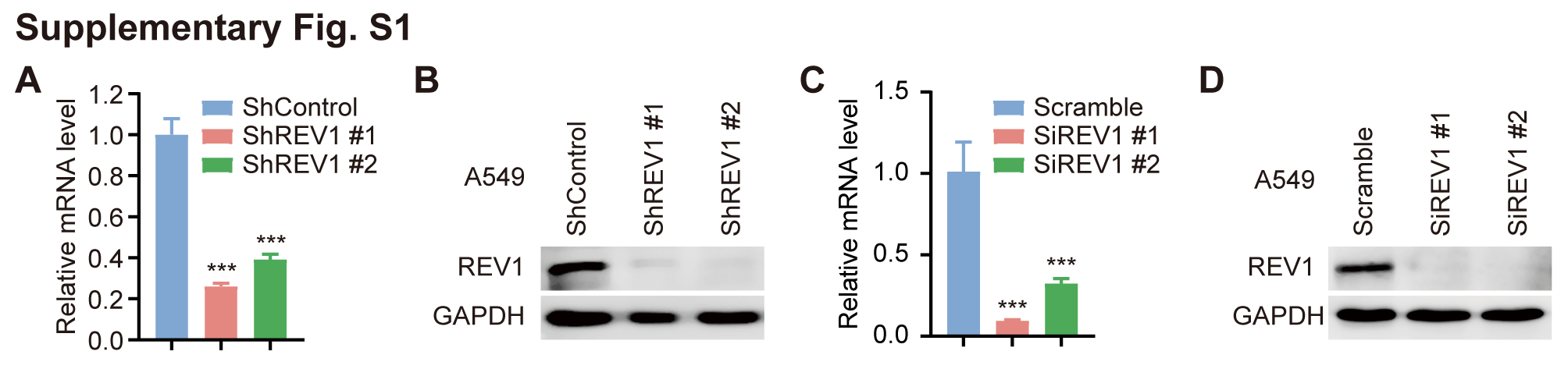

Supplement: Supplementary file 1 — Supplementary Material 1: Fig. S1. REV1 expression can be effectively knocked down by shRNAs and siRNAs. A The mRNA levels of the indicated molecules were measured by qRT-PCR in shControl and shREV1 lung cancer cells. *** P < 0.001 (n = 4). B REV1 knockdown cell line was successfully constructed by using shRNA technology (n = 3). C The mRNA levels of the indicated molecules were measured by qRT-PCR in scramble and siREV1 lung cancer cells.*** P < 0.001 (n = 4). D A549 cells transfected with the indicated siRNAs were harvested and analyzed by Western blotting (n = 3). [file 12929_2024_1044_MOESM1_ESM.tif]

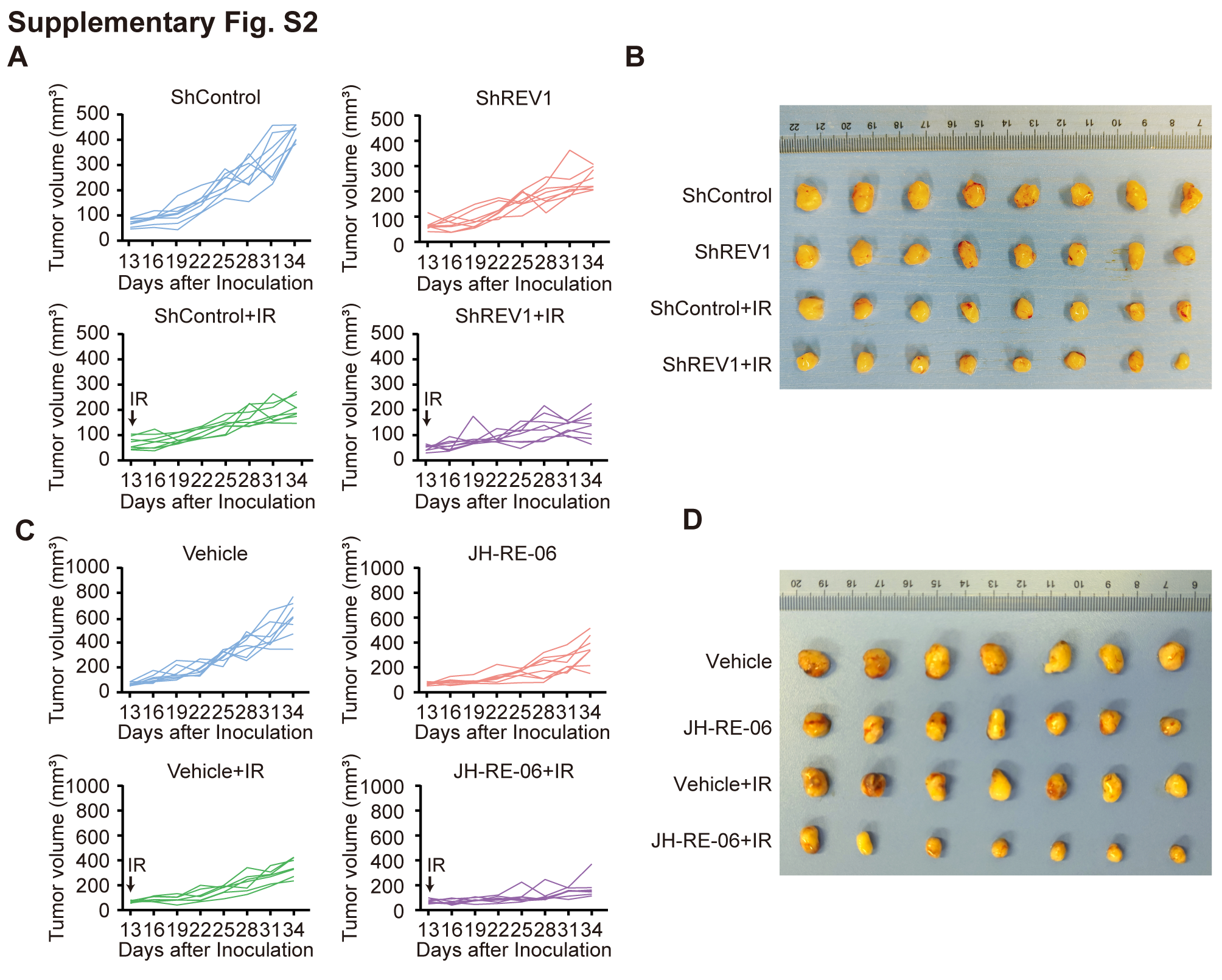

Supplement: Supplementary file 2 — Supplementary Material 2: Fig. S2. Targeting REV1 enhances the radiosensitivity of lung cancer cells in vivo. A REV1 was inhibited by shRNA knockdown and then combined with radiotherapy. Growth curves were plotted for each mouse (n = 8). B Photographs of transplanted tumors from the indicated groups (n = 8). C Growth curves for each mouse in JH-RE-06 combined with radiotherapy experiments were presented (n = 7). D Pictures of xenograft tumors from the corresponding groups (n = 7). [file 12929_2024_1044_MOESM2_ESM.tif]

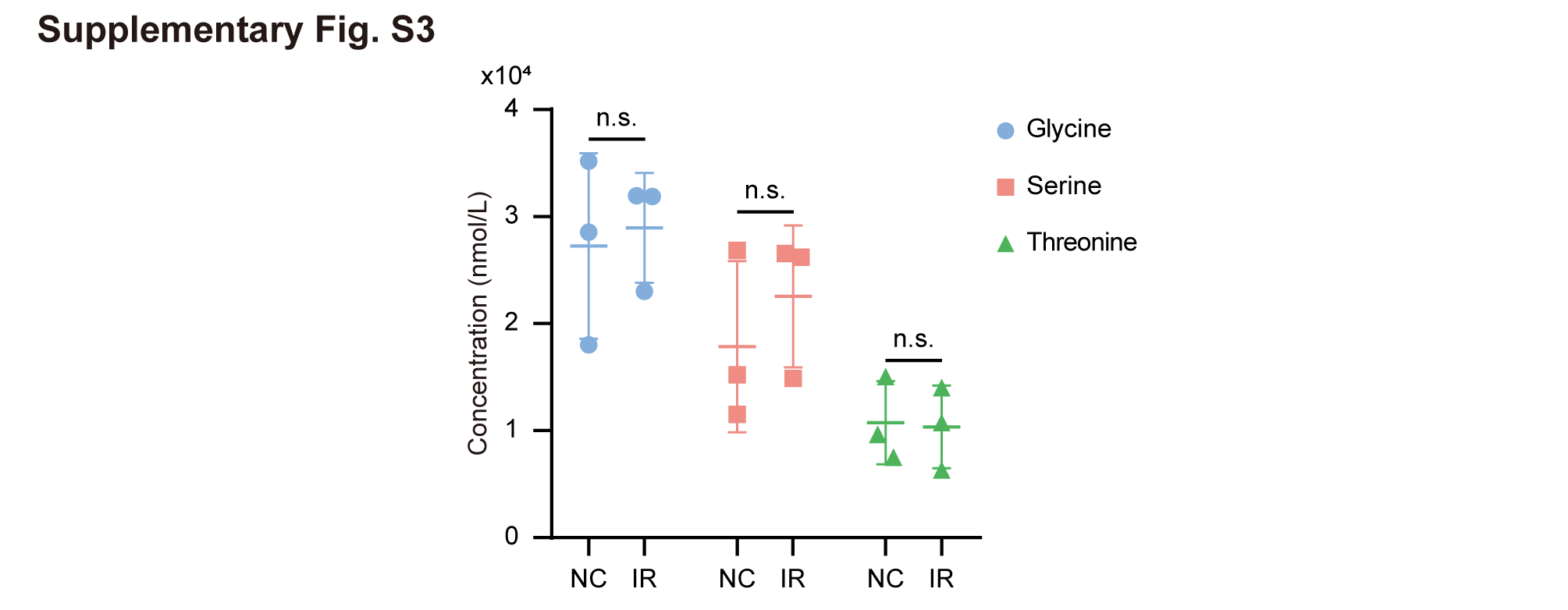

Supplement: Supplementary file 3 — Supplementary Material 3: Fig. S3. Radiotherapy had little effect on glycine, serine, and threonine metabolism. The levels of glycine, serine, and threonine in the control and radiotherapy group were determined by LC-MS. n.s. P > 0.05 (n = 3). [file 12929_2024_1044_MOESM3_ESM.tif]

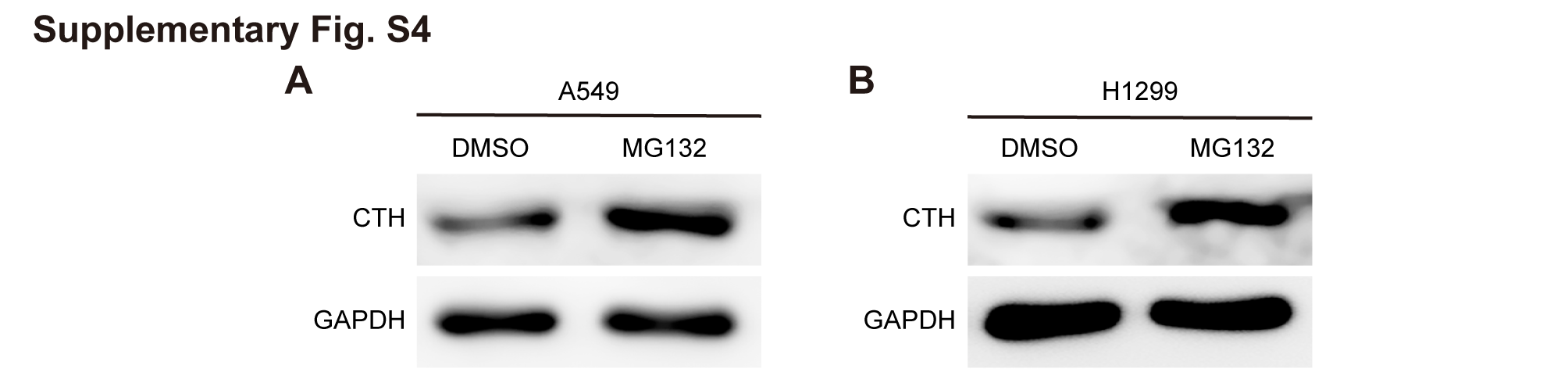

Supplement: Supplementary file 4 — Supplementary Material 4: Fig. S4. CTH is regulated by the ubiquitin-proteasome system. A549 cells (A) and H1299 cells (B) were treated with DMSO or MG132 for 4 h before protein extraction. The CTH expression was detected by Western blotting (n = 3). [file 12929_2024_1044_MOESM4_ESM.tif]

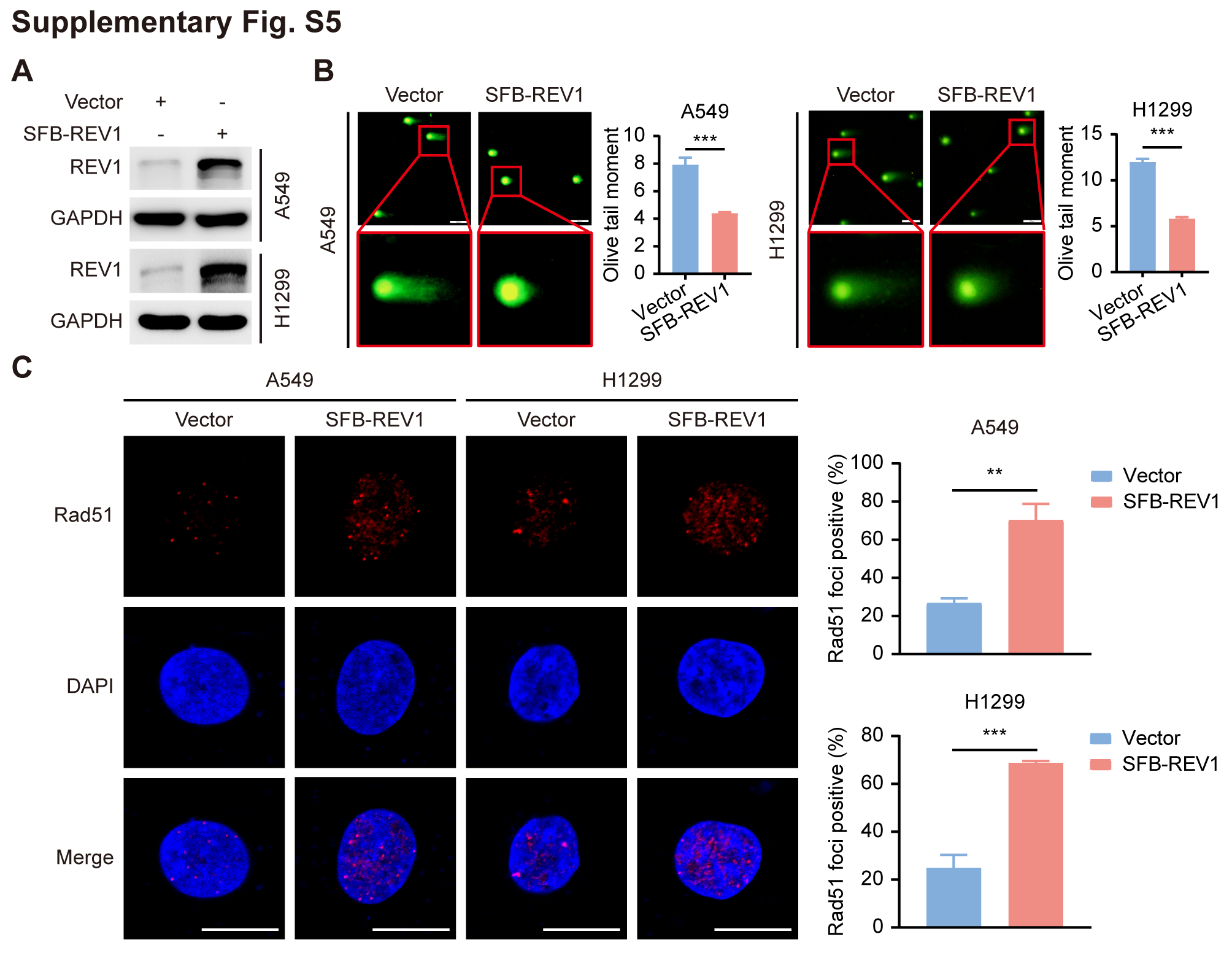

Supplement: Supplementary file 5 — Supplementary Material 5: Fig. S5. Overexpression of REV1 reduces lung cancer radiosensitivity in vitro. A A549 and H1299 cells were transfected with vector and SFB-REV1 and collected for Western blotting (n = 3). B A549 and H1299 cells transfected with vector and SFB-REV1 were subjected to comet assay after receiving 6 Gy irradiation, representative images and the statistics of comet tail moment are shown. *** P < 0.001 (n = 100). C A549 and H1299 cells transfected with vector and SFB-REV1 were subjected to Rad51 immunofluorescence staining after receiving 6 Gy irradiation. The proportion of Rad51 foci-positive cells was calculated under a fluorescence confocal microscope. ** P < 0.01, *** P < 0.001 (n = 3). [file 12929_2024_1044_MOESM5_ESM.tif]
