# Supplementary material for: USP9X-mediated REV1 deubiquitination promotes lung cancer radioresistance via the action of REV1 as a Rad18 molecular scaffold for cystathionine γ-lyase
Source: J Biomed Sci. 2024 May 28;31:55. doi: 10.1186/s12929-024-01044-3 (PMC11131313; doi:10.1186/s12929-024-01044-3)
Supplement: Supplementary file 6 — Supplementary Material 6: Table S1. Sequences of siRNA and shRNA used in this study. Table S2. Sequences of primers used for Real-time quantitative PCR. [file 12929_2024_1044_MOESM6_ESM.docx]

**Supplementary Tables**

| **Genes** | **Sequences** | **Source** |
| --- | --- | --- |
| Scramble | 5′-UUCUCCGAACGUGUCACGUTT-3′ | Chen et al., 2022 |
| SiREV1 | #1: 5′-GAACAGUGACGCAGGAAUA-3′ | Chen et al., 2022 |
|  | #2: 5′-GCAUCAAAGCUGGACGACU-3′ |  |
| SiRad18 | #1: 5′-CCAGCCAAAUCUCCUGCUUTT-3 | Chen et al., 2022 |
|  | #2: 5′-GCGUCUUGAAGCUAGUAAATT-3′ |  |
| Control | 5′-CCTAAGGTTAAGTCGCCCTCG-3′ | Jie et al., 2022 |
| ShREV1 | #1: 5′-AGCTCAACAACAGGGTAAATC-3′ | This study |
|  | #2: 5′-TATCAGTGGGTATACGAAATA-3′ |  |

**Table S1.** Sequences of siRNA and shRNA used in this study.

| **Genes** | **Sequences (5'--3')** |
| --- | --- |
| CTH | F: GGCCTGGTGTCTGTTAATTGT |
|  | R: GCCATTCCGTTTTTGAAATGCT |
| REV1 | F: CAGTCGCCCATCAGTTCAGT |
|  | R: CTTTGTGCTCCTCTTCGGTG |
| GAPDH | F: GAGTCAACGGATTTGGTCGT |
|  | R: GACAAGCTTCCCGTTCTCAG |

F, forward primer; R, reverse primer.

**Table S2.** Sequences of primers used for Real-time quantitative PCR.
